# Longitudinal trends in malaria testing rates in the face of elimination in eastern Myanmar: a 7-year observational study

**DOI:** 10.1186/s12889-021-11749-x

**Published:** 2021-09-22

**Authors:** Jade D. Rae, Jordi Landier, Julie A. Simpson, Stéphane Proux, Angela Devine, Richard J. Maude, Aung Myint Thu, Jacher Wiladphaingern, Ladda Kajeechiwa, May Myo Thwin, Saw Win Tun, François H. Nosten

**Affiliations:** 1 Shoklo Malaria Research Unit (SMRU), Mahidol-Oxford Tropical Medicine Research Unit (MORU), Faculty of Tropical Medicine, Mahidol University, Mae Sot, Thailand; 2 Mahidol-Oxford Tropical Medicine Research Unit (MORU), Faculty of Tropical Medicine, Mahidol University, Bangkok, Thailand; 3 Centre for Tropical Medicine and Global Health, Nuffield Department of Medicine, University of Oxford, Oxford, UK; 4 IRD (Institut de Recherche pour le Developpement), Aix Marseille Univ, INSERM, SESSTIM, Marseille, France; 5 Centre for Epidemiology and Biostatistics, Melbourne School of Population and Global Health, The University of Melbourne, Melbourne, Australia; 6 Global and Tropical Health Division, Menzies School of Health Research, Charles Darwin University, Darwin, Australia; 7 Harvard TH Chan School of Public Health, Harvard University, Boston, USA; 8 The Open University, Milton Keynes, UK

**Keywords:** Malaria, *P. falciparum*, *P. vivax*, Elimination, Community health worker, Testing rate, RDT, Integrated health services

## Abstract

**Background:**

Providing at-risk communities with uninterrupted access to early diagnosis and treatment is a key component in reducing malaria transmission and achieving elimination. As programmes approach malaria elimination targets it is critical that each case is tested and treated early, which may present a challenge when the burden of malaria is reduced. In this paper we investigate whether malaria testing rates decline over time and assess the impacts of integrating malaria and non-malaria services on testing rates in the malaria elimination task force (METF) programme in the Kayin state of Myanmar.

**Methods:**

A retrospective analysis was conducted using weekly collected data on testing rates from a network of more than 1200 malaria posts during the period from 2014 to 2020. To determine whether monthly testing rates changed over the years of programme operations, and whether integrating malaria and non-malaria services impacted these testing rates, we fitted negative binomial mixed-effects regression models to aggregate monthly data, accounting for malaria seasonal variation.

**Results:**

In the first year of malaria post operation, testing rates declined, correlating with a decline in attendance by people from outside the malaria post catchment area, but then remained fairly constant (the Rate Ratio (RR) for 2nd versus 1st year open ranged from 0.68 to 0.84 across the four townships included in the analysis, the RR for 3rd to 6th year versus 1st year open were similar, ranging from 0.59–0.78). The implementation of a training programme, which was intended to expand the role of the malaria post workers, had minimal impact on testing rates up to 24 months after training was delivered (RR for integrated versus malaria-only services ranged from 1.00 to 1.07 across METF townships).

**Conclusion:**

Despite the decline in malaria incidence from 2014 to 2020, there has been no decline in the malaria testing rate in the METF programme after the establishment of the complete malaria post network in 2016. While the integration of malaria posts with other health services provides benefits to the population, our evaluation questions the necessity of integrated services in maintaining malaria testing rates in areas approaching elimination of malaria.

**Supplementary Information:**

The online version contains supplementary material available at 10.1186/s12889-021-11749-x.

## Background

Despite the substantial progress made in reducing the incidence and prevalence of malaria since 2000 [1, 2], achieving malaria elimination remains challenging. Globally, progress towards elimination has stalled [3], and additional efforts must be made to reach the WHO elimination goals.

In the Greater Mekong Subregion, the goal is to eliminate malaria rapidly to prevent the spread of artemisinin resistant *Plasmodium falciparum* (*P. falciparum*). Successful elimination can be achieved with existing diagnostics [4, 5] and interventions [6–10]. However, the effectiveness of these resources relies on their uninterrupted availability and community utilisation [11, 12]. To meet these requirements, malaria elimination programmes must rely on village-based malaria posts operated by trained malaria post workers. The aim of these malaria posts is to diagnose cases early, and to provide treatment within the first 48 h of malaria induced fever, thus preventing onward transmission, and progression to severe malaria [13, 14].

Early testing and treatment of malaria is essential, particularly in the elimination phase where each undetected case threatens elimination efforts at the village level [15]. As the incidence of malaria declines, community usage of malaria posts may also decline as reported in malaria posts operating in the Mon State, and the Kyainseikgyi township of the Kayin State of Myanmar [16]. In these malaria posts, providing malaria post workers with skills in identifying and referring cases of other diseases (including respiratory illness, diarrhoea and tuberculosis) resulted in an increase in community acceptance of the programme, and an immediate and dramatic increase in malaria testing rates in some of the malaria post cohorts [16].

In the absence of additional studies, the impact of declining incidence and community acceptance of malaria-only services on malaria testing rates remains inconclusive, and additional studies are needed to develop recommendations on how to maintain testing rates for malaria elimination programmes globally.

Using data collected from the Malaria Elimination Task Force (METF) programme in the Kayin State of Myanmar, this study has three primary objectives: first, to investigate the temporality of malaria testing rates in relation to declining malaria incidence; second, to assess the impact of expanding the malaria post workers’ role on these testing rates; and third, to investigate changes in testing quality over time.

## Methods

### Study design and setting

We conducted a retrospective observational study of 593,186 malaria rapid diagnostic tests conducted between 2014 and 2020 in a cohort of 1250 malaria posts supported by the METF programme and evaluated the temporal trends and the impact of integrated community health worker training on testing rates.

**Fig. 1:**
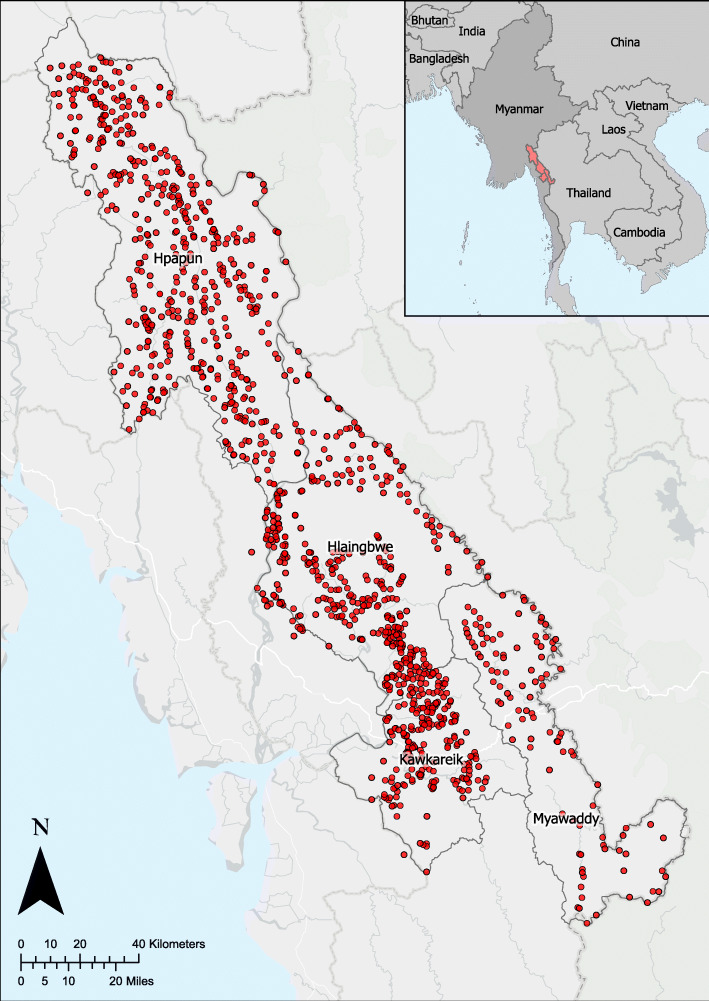
The Malaria Elimination Task Force malaria post network in the Kayin State of Myanmar. Each red point corresponds to a malaria post operated by a malaria post worker, trained to deliver uninterrupted access to diagnosis and treatment of malaria. The Kayin State is divided into 4 townships: Hpapun, Hlaingbwe, Kawkareik, and Myawaddy. Map generated using ArcGIS version 2.5

The Kayin State of Myanmar is located on the border of Myanmar and Thailand, approximately 150 kms south of Myanmar’s capital, Naypyidaw, and 150kms east of Yangon. The METF programme operates in 4 townships of the Kayin State of Myanmar: Hpapun, Hlaingbwe, Kawkareik and Myawaddy (Fig. 1). Hpapun, in the North, is mountainous and forest dense, and there are typically two annual malaria transmission peaks, coinciding with the wet season from May to October, and the cold season from December to January. Hlaingbwe, south of Hpapun, is geographically flatter except for the Dawna range which divides it. Villages in Kawkareik and Hlaingbwe townships on West of the Dawna range have greater access to other health infrastructures and road networks that allow for easier navigation compared with the villages in Myawaddy township in the East, where the geography is more mountainous. In Hlaingbwe, Kawkareik and Myawaddy there is typically one malaria transmission peak in June, coinciding with wet season.

### Malaria posts and passive case detection

The METF programme began in 2014 in response to a high incidence of *P. falciparum* and *P. vivax* malaria in the Kayin State, a high prevalence of sub-microscopic carriers in some villages, and the ongoing threat posed by antimalarial drug resistance in the Greater Mekong Subregion [17].

During the period from the commencement of the programme in 2014 to 2016, the METF malaria post network expanded to 1250 malaria posts, covering an estimated 18,002km^2^ and serving an estimated population of 350,000. These malaria posts are operated by malaria post workers, trained to deliver uninterrupted access to free early diagnosis and treatment of malaria to all people who present to the malaria post with fever [18]. Of these malaria posts, 42 are based within a clinic or a primary health care facility with resources to manage non-malaria fever and offering inpatient department (IPD) services [19].

Fever cases that present to the malaria post are tested using a *P. falciparum - P. vivax* rapid diagnostic test (RDT) (SD Bioline P. f/P. v, Standard Diagnostics/Alere Republic of Korea). In the Kayin State of Myanmar, the high prevalence of glucose-6-phosphate dehydrogenase (G6PD) deficiency has limited the ability to safely deliver the necessary radical cure to those infected with *P. vivax* in this region [20–22]. Treatment regimens for *P. falciparum* and *P. vivax* in the METF programme are provided in detail elsewhere [14, 18].

An initial 5-day training programme was provided to all METF malaria post workers. After opening, malaria post worker knowledge and skills have been assessed twice a year. If needed, refresher training is organised to address issues identified, ensuring malaria posts remain functional.

At the opening of each malaria post, community engagement occurs at the township, district, and village levels to provide information on the METF programme, the role of the malaria post, and the importance of early diagnosis and treatment [23–25].

### Data collection and management

#### Surveillance data

Each week, the malaria post workers send a summary of the number of fever cases, and the number of RDT-confirmed *P. falciparum* and *P. vivax* infections by age and gender. Data are available within 21 days of the start of reporting week for the malaria posts in Hpapun, which rely on paper data transmission to the nearest data entry site due to the absence of reliable cell phone network coverage. At the data entry site in Hpapun, weekly data are entered and emailed to the central METF team in Mae Sot, Thailand. Data are available within 14 days for the malaria posts in Hlaingbwe, Kawkareik and Myawaddy, where access to a cell phone network is available and data are sent via SMS. Each week, data from all four townships are combined with data from the previous weeks using Microsoft Access, resulting in the complete weekly database containing all surveillance data collected over the programme. Data are checked and cleaned each week and any errors in data entry are corrected after communication with the data entry team who review the raw data [18].

#### Integrated community malaria worker (ICMW) training data

From February 2019, an ICMW training programme was rolled out in some malaria posts in Hlaingbwe, Kawkareik and Myawaddy. Training was conducted over a three-day period, with the aim to provide malaria post workers with skills in recognising, and referring to a health centre, patients with suspected leprosy, tuberculosis (TB), human immunodeficiency virus (HIV), filariasis, sexually transmitted infections (STIs), or dengue. In addition to training, the ICMWs were provided with basic first aid supplies, multivitamins, and oral rehydration salts. Data are entered and maintained using Microsoft Excel.

#### RDT quality control data

Since 2016, RDT quality control has been performed monthly on RDTs from a random subset of 20 malaria posts. The indicators used for sub-optimal RDT quality were classified into three subgroups based on the source of quality issue: physical damage, record management, and user-related issues. A detailed description of quality control assessment methods is provided in more detail elsewhere [26]. Data are entered and maintained using SPSS (version 27).

### Statistical analysis

Incidence and RDT rates (per 1000 persons per month) were calculated from weekly malaria post reports. For each month or month open, the incidence was calculated as the number of cases over person-time exposed at the malaria post, and RDT rates were calculated as the number of RDTs completed over person-time exposed at the malaria post. Incidence and RDT rates were then averaged over each township.

The denominator (person-time exposed) was calculated at the malaria post level using village census information collected at malaria post opening.

To estimate the relative change in RDT rates for each additional year of malaria post operation, negative binomial mixed-effects modelling was performed with the population at each malaria post per month as the person-time denominator. A separate model was used for each township to allow for differing malaria seasonality and transmission dynamics. To account for village-level differences in RDT rates, a random intercept was included for malaria posts, and to account for changes in temporal patterns between malaria posts, a random slope was included for the number of years they had been open. To account for malaria seasonality, Fourier terms were included in the regression model, being a combination of sine and cosine functions. Each pair of Fourier terms was fitted, with the optimal Fourier terms selected based on the Akaike Information Criterion (AIC). To account for differences in testing rates between clinic-based and non-clinic-based malaria posts, a covariate for clinic status was included in the model.

To investigate the potential impact of ICMW training on testing rates, the same negative binomial mixed-effects modelling was performed with the inclusion of a random slope for ICMW training period in place of years open. Malaria posts included in this analysis were limited to those with at least 12 months follow up post-ICMW training to capture a complete seasonal transmission cycle.

The data collected during RDT quality control were summarised by year and township to investigate the temporal and spatial patterns of quality control results.

All statistical analyses were performed in R (version 3.6) and mapping performed in ArcGIS Pro (version 2.5).

## Results

### Temporality of malaria testing rates

**Fig. 2:**
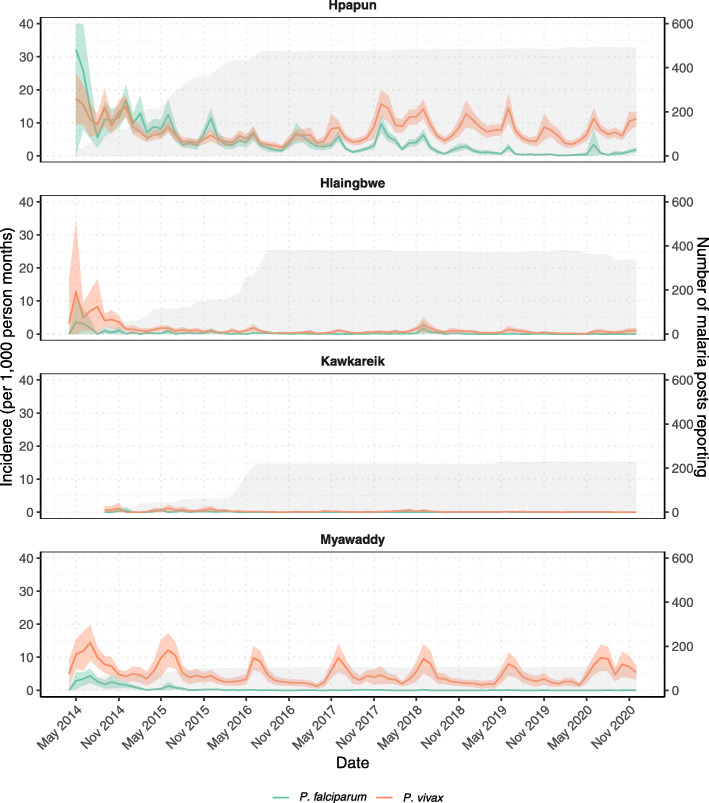
Average monthly *P. falciparum* and *P. vivax* incidence at malaria posts by township. Monthly incidence of *P. falciparum* (green line), with 95% confidence intervals (green area) and *P. vivax* (orange line), with 95% confidence intervals (orange area) calculated at the malaria post level, averaged over township. Total number of malaria posts providing weekly reports shown in grey. Upper confidence interval for *P. falciparum* incidence rate in Hpapun capped at 40

Since the start of the METF programme in 2014, the incidence of *P. falciparum* has declined dramatically in all townships (Fig. 2). For malaria posts in Hpapun, where the burden of malaria is highest, the yearly *P. falciparum* incidence in 2020 had declined by 94% from the start of the programme in 2014, and by 78% from when the complete malaria post network was functioning in 2016. The same declines have not been observed for *P. vivax* incidence (Fig. 2). An additional figure with different y-axis scales provides another way of viewing incidence trends [see Additional file 1, Fig. S1]. An additional table provides more details on the average monthly incidence by malaria post [see Additional file 2, Table S1].

According to the data, the delay between fever onset and malaria RDT was less than two days for most consultations across townships and programme years. For the period of 2014 to 2020, between 72 to 83% of consultations occurred within 48 h of fever onset, 10 to 16% within 2 to 3 days, and 6 to 11% after 3 days of fever onset.

**Fig. 3:**
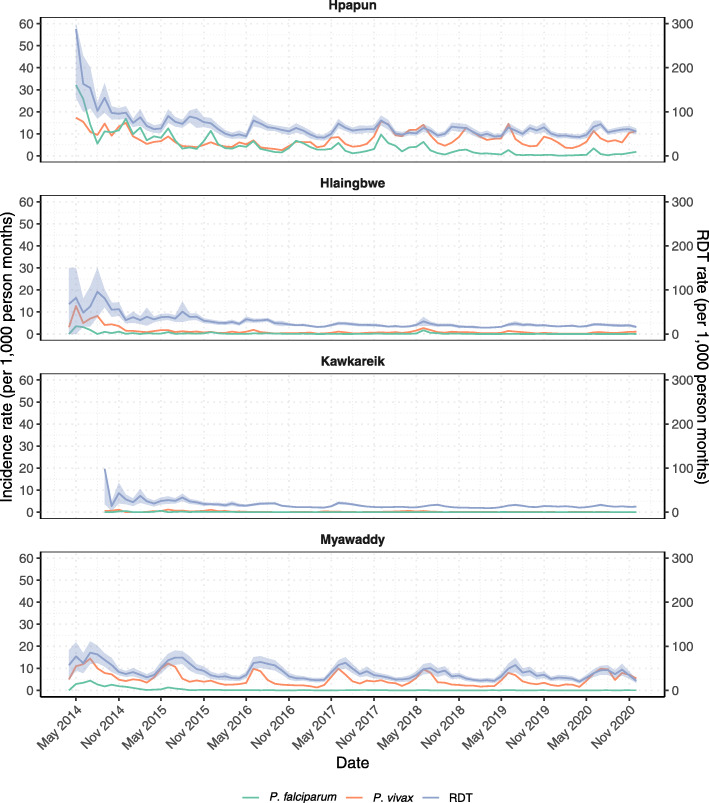
Average monthly rate of RDTs, and malaria incidence by date and township. Average rapid diagnostic testing rate (RDT – purple line), with 95% confidence intervals (purple area), and average *P. falciparum* (green line) and *P. vivax* (orange line) incidence rates in the METF malaria posts by date. Upper confidence interval for RDT rate in Hpapun capped at 300

From 2014 to 2016, RDT rates declined, a result of the establishment and expanding METF malaria post network. From 2016, with a network of more than 1200 malaria posts, the RDT rates stabilised - a reflection of more localised malaria post services (Fig. 3). An additional figure with different y-axis scales provides another way of viewing trends in incidence by date [see Additional file 1, Fig. S2]. In 2020, the median annual RDT rate per village was 44% (Interquartile range (IQR): 21–74%) in Hpapun, 16% (IQR: 8–27%) in Hlaingbwe, 13% (IQR: 7–20%) in Kawkareik, and 29% (IQR: 14–56%) in Myawaddy. An additional table provides more details on the average monthly RDT rates by township from 2014 to 2020 [see Additional file 2, Table S1].

**Fig. 4:**
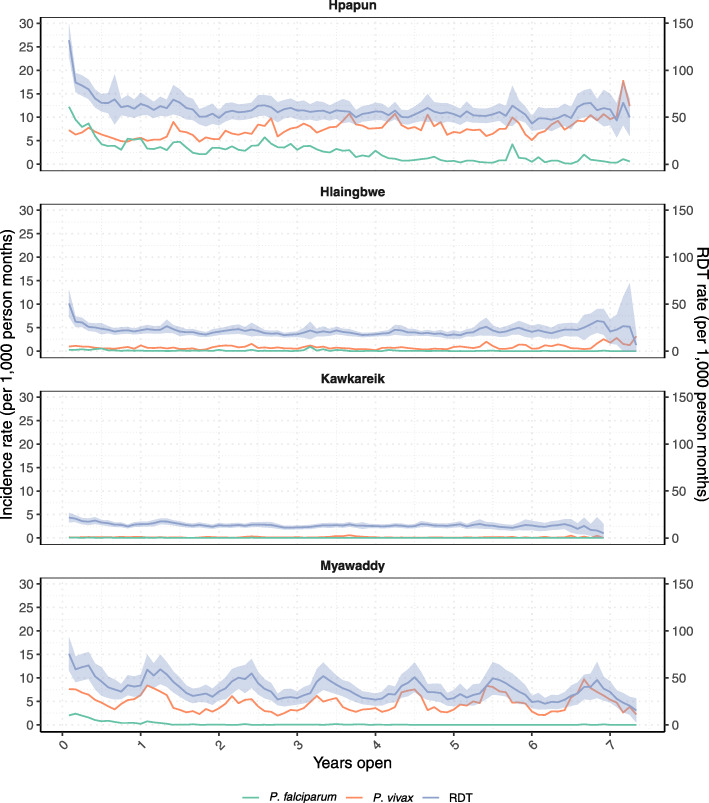
Average monthly rate of RDTs, and malaria incidence by malaria post time open. Average rapid diagnostic testing rate (RDT – purple line), with 95% confidence intervals (purple area), and average *P. falciparum* (green line) and *P. vivax* (orange line) incidence rates in the METF malaria posts by years open

In Fig. 4, which shows the average monthly RDT rate and incidence of *P. falciparum* and *P. vivax,* as a function of the number of years the malaria post has been opened, we observed no decline in RDT rates after the first few months of malaria post opening. An additional figure with different y-axis scales provides another way of viewing trends in incidence by malaria post time open [see Additional file 1, Fig. S3]. An additional table provides more details on the average monthly RDT rates and incidence by number of malaria post years open [see Additional file 2, Table S2].

The estimated rate ratios from the regression model confirm the trend seen in Fig. 4. An initial decrease in RDT rates from the first to second year of malaria post opening was significant across all four townships, shown by the decrease in the estimated rate ratios, ranging from 0.68 to 0.84-fold reduction (Table 1). After the first year open, there was no significant difference in the RDT rates between years, as shown by the relatively stable rate ratios and overlapping 95% confidence intervals.

**Table 1:** Relative change in monthly RDT rates, by township and year open. RDT rates measured at the malaria post level

Covariate	Hpapun;	Hlaingbwe;	Kawkareik;	Myawaddy;
	RR (95% CI)	RR (95% CI)	RR (95% CI)	RR (95% CI)
Year open 1	Reference	Reference	Reference	Reference
2	0.68 (0.65, 0.72)	0.77 (0.74, 0.80)	0.84 (0.79, 0.90)	0.78 (0.72, 0.84)
3	0.68 (0.64, 0.71)	0.73 (0.69, 0.76)	0.70 (0.65, 0.76)	0.71 (0.64, 0.78)
4	0.64 (0.60, 0.68)	0.76 (0.71, 0.80)	0.78 (0.72, 0.85)	0.68 (0.60, 0.76)
5	0.63 (0.59, 0.67)	0.71 (0.66, 0.76)	0.66 (0.59, 0.74)	0.64 (0.57, 0.72)
6	0.64 (0.60, 0.69)	0.64 (0.59, 0.70)	0.59 (0.52, 0.66)	0.59 (0.51, 0.67)
7	0.74 (0.67, 0.82)	0.57 (0.48, 0.67)	0.46 (0.25, 0.85)	0.54 (0.47, 0.64)
Clinic-based				
No	Reference	Reference	Reference	Reference
Yes	1.81 (1.31, 2.52)	3.20 (1.45, 7.05)	1.46 (0.70, 3.02)	1.17 (0.65, 2.10)

Mixed effects negative binomial model with random intercept for malaria post and random slope for years open

Seasonality captured using 3 Fourier terms per year

*RDT* Rapid diagnostic test; *RR* rate ratio; *CI* confidence interval

Clinic-based malaria posts had higher RDT rates than malaria posts not based within a clinic in both Hpapun (Rate Ratio (RR) = 1.81, 95% CI: 1.31, 2.52) and Hlaingbwe (RR = 3.20, 95% CI: 1.45 7.05). The inclusion of an interaction term between year open and clinic status was insignificant in all townships, which suggests the relationship between RDT rate and year open is not dependent on whether a malaria post is based within a clinic. An additional figure is provided for the model-based predictions of RDT rates shown in Table 1 [see Additional file 3, Fig. S4].

### Integrated community malaria worker (ICMW) training

In 2019, an ICMW training programme was provided to 61% (237/387) of malaria posts in Hlaingbwe, 65% (152/232) in Kawkareik, and 43% (46/107) in Myawaddy. This training was provided at different times during 2019, resulting in varying follow-up times. Malaria posts with at least 12-months follow up were included in the analysis and accounted for 198 malaria posts in Hlaingbwe, 150 in Kawkareik and 46 in Myawaddy.

The estimated rate ratios from the regression model, showed minimal or no increases in RDT rates after ICMW training in Hlaingbwe (RR = 1.07, 95% CI: 1.00–1.15), Kawkareik (RR = 1.00, 95% CI: 0.91, 1.10), and Myawaddy (RR = 1.02, 95% CI: 0.89, 1.17) (Table 2).

**Table 2:** Relative change in monthly RDT rates after ICMW-training by township. RDT rates measured at the malaria post level

Covariate	Hlaingbwe;	Kawkareik;	Myawaddy;
	RR (95% CI)	RR (95% CI)	RR (95% CI)
Training period			
Pre-ICMW	Reference	Reference	Reference
Post-ICMW	1.07 (1.00, 1.15)	1.00 (0.91, 1.10)	1.02 (0.89, 1.17)
Clinic-based			
No	Reference	Reference	Reference
Yes	4.60 (1.79, 11.82)	1.33 (0.35, 5.11)	0.93 (0.38, 2.19)

Mixed effects negative binomial model with random intercept for malaria post and random slope for before and after

ICMW training. Seasonality captured using 3 Fourier terms per year

Pre-ICMW training period: 24 months; Post-ICMW training period: ≤ 16 months in Hlaingbwe, ≤ 24 months in Kawkareik, and ≤ 19 months in Myawaddy

*RDT* Rapid diagnostic test; *ICMW* Integrated community malaria worker; *RR* rate ratio; *CI* confidence interval

**Fig. 5:**
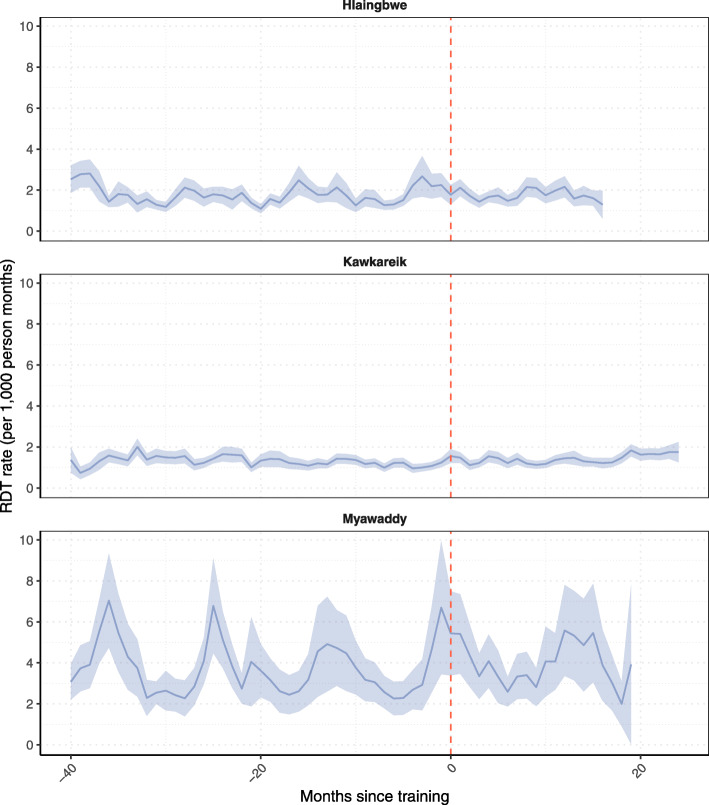
Average monthly rate of RDTs in malaria posts that received integrated community health worker training. Average rapid diagnostic testing rate (RDT – purple line), with 95% confidence intervals (purple area) before and after training was delivered (red line) by months since training

Figure 5 shows the comparison of RDT rates before and after ICMW training, with no observable impact of ICMW training on RDT rates up until 16 months after training was delivered to malaria posts in Hlaingbwe, 24 months for malaria posts in Kawkareik, and 19 months for malaria posts in Myawaddy (Fig. 5).

### RDT quality control

From 2016 to 2020, RDT quality control was conducted on a random subset of 11,598 RDTs from 706 METF malaria posts. The percentage of RDTs that were damaged during storage or transport, resulting in dirty or damaged test strip, was small (0–8%) across programme years and townships, and decreased overtime in Hpapun and Myawaddy. Recording of RDT results by the malaria post workers improved over time in all townships, accounting for 10 to 17% of RDTs checked in 2016, compared to 1 to 7% of RDTs checked in 2020. RDTs classified as having user-related issues were those with blood on the side of the well (difficulty loading blood onto RDT), or where the blood did not migrate, but a test result was recorded. These problems decreased over time except for in Myawaddy, where the number of RDTs checked in 2020 was small (*n* = 75). An additional file provides more details on the quality control results [see Additional file 4, Table S3].

Post-diagnosis validation of RDT results has not been possible due to fading of the control line and/or blood backflow along the test strip over time. The median delay between RDT performance and quality control was 89 days (IQR = 66–124) in Hpapun, 83 days (IQR = 62–116) in Hlaingbwe, 82 days (IQR = 56–108) in Kawkareik, and 75 days (IQR = 57–100) in Myawaddy.

## Discussion

The backbone of a successful malaria elimination programme is its ability to provide access to early diagnosis and treatment of malaria cases. This retrospective analysis of RDT rates from malaria posts in the METF programme demonstrates that maintaining testing rates is possible, even when approaching elimination targets, and despite the benefits of integrating malaria with non-malaria services, this integrated healthcare approach may not influence malaria testing rates.

In the first two years of the METF programme, from 2014 to 2016, the malaria post network expanded. When the complete METF network of 1250 malaria posts was established, the more widespread, localised availability of diagnosis and treatment resulted in a reduction in testing rates at the malaria posts, which had once provided services to people from within and outside of their targeted communities.

RDT rates also declined in the first year of malaria post opening, a result of a higher attendance in the first months of malaria post opening, particularly in locations where existing health infrastructure was limited. Following the first year of malaria post operation, there was no further decline in RDT rates with increasing malaria post years open across the townships. Malaria posts that were based within a clinic had higher RDT rates than non-clinic-based malaria posts in Hpapun and Hlaingbwe, which suggests a higher attendance to these malaria posts, irrespective of number of years open. While population surveys could reveal more insight into these findings, the likely explanation for higher attendance at these malaria posts is their ability to provide a wider range of services when compared with non-clinic-based malaria posts.

The differences in RDT rates between townships is explained by the differences in malaria incidence. Hpapun has a higher incidence of *P. falciparum* and *P. vivax*, and Myawaddy has a higher incidence of *P. vivax* and therefore there are more cases of fever which present to the malaria posts seeking a malaria test in Hpapun and Myawaddy than in Hlaingbwe and Kawkareik.

Another indicator of malaria post service uptake is the delay between fever onset and malaria post consultation. In the METF malaria posts, the majority of consultations occurred in the first 48 h of fever in all townships and programme years, which means the majority of consultations were conducted before positively diagnosed individuals became infectious, thereby reducing onward malaria transmission [13].

The result of sustained RDT rates has been the continued effectiveness of early diagnosis and treatment, and the ongoing decline in *P. falciparum* incidence in the METF programme. The impact on *P. vivax* has been less dramatic due to the necessity of radical cure to eliminate hypnozoites, the dormant liver stages of infection that trigger relapses. Radical cure involves treating the blood stage of infection and combining it with an antimalarial active against hypnozoites, where the latter presents a risk of haemolysis in G6PD deficient individuals [27]. In the Kayin State of Myanmar, the prevalence of G6PD deficiency is high and presents a challenge to the safe administration of radical cure [20, 21, 27]. Sensitive point-of-care testing for G6PD deficiency must be available to allow for the safe administration of these drugs.

In 2019, an ICMW training programme was rolled out in some malaria posts in Hlaingbwe, Kawkareik and Myawaddy. This ICMW training package resulted in a 7% increase in RDT rates in Hlaingbwe, but with a wide confidence interval containing the possibility of no impact (95% CI: 0–15%). In Kawkareik and Myawaddy townships there was no evidence of ICMW training impact on RDT rates.

Despite the importance of maintaining RDT rates in malaria elimination programmes, there exists only one comparative study on RDT rates over time which is the study conducted at the Medical Action Myanmar (MAM) malaria posts in the Mon and Kayin (Kyainseikgyi township) States of Myanmar. This study concluded that ICMW training was associated with an increase of between 1.10 and 5.4-fold in RDT rates which had previously declined over time [16].

There are key differences between the MAM and METF programs which could explain this contrasting result. First, the details of the training outlined by MAM suggest a more intensive, long-term training programme compared to the three-day training programme rolled out by METF [16]. The cost of referral, and transport to and from the hospital were also provided in the MAM programme. However, this does not explain the differences in the impact of ICMW training on RDT rates between the cohorts included in their analysis, assuming the same ICMW training programme and supplies are provided to all MAM malaria posts [16].

Second, the METF conducted routine monitoring and evaluation visits and refresher trainings for basic malaria post activities, as well as ongoing community engagement activities. These activities are essential in ensuring a high quality of service delivery and in maintaining community acceptance and utilisation of these services [24–26]. It is possible that a key aspect of maintaining RDT rates is providing support to the malaria post worker in their task, whether basic or advanced. The METF programme demonstrates it is possible to maintain RDT rates in the face of declining malaria incidence through ongoing community engagement.

For the ICMW training programme to be effective, we need to consider the challenges faced by community health workers, what additional tasks can reasonably be delegated to these workers, and how best to support them in performing these additional tasks [28, 29]. While a broad training programme in many non-malaria illnesses may sound appealing, providing knowledge and skills in locally prevalent illnesses may be more effective. In addition, the provision of referral costs for patients may be a necessary component in the ICMW framework, particularly in remote areas where referral to the nearest health facility, without providing transport, may hinder the impact of these non-malaria services.

A limitation of this study is that these results may be context specific, where demographic and geographic factors likely play a role in how these populations interact with malaria post services. Additional studies of RDT rates in malaria elimination programmes globally will provide more insight in how malaria testing rates should be maintained in varying contexts. In addition, village population numbers used to calculate the incidence and RDT rates were collected at malaria post opening and have not been updated. Annual village censuses would improve the accuracy of these calculations.

## Conclusion

Despite the substantial declines in the incidence of *P. falciparum* since the commencement of the METF programme in 2014, there has been no decline in RDT rates since the complete METF malaria post network was established in 2016. This is likely a result of the continued community engagement, and routine monitoring and evaluation of malaria post performance which ensures continued acceptance of these services, and a high quality of service delivery, both of which should be considered essential to malaria elimination efforts.

The integration of malaria testing and treatment with other health services promotes the development of complementary skills in the malaria post workers who are trusted by the communities they serve, allowing these populations to receive benefits beyond those targeted to malaria. However, in the METF programme the ICMW training package has had minimal impact on malaria RDT testing rates, and alternative reasons for reduced community attendance should be investigated in areas where RDT rates have declined.

## Supplementary Information

**Additional file 1.** Incidence and RDT rate figures: Additional figures on incidence and RDT rates by date and malaria post time open.

**Additional file 2. **Monthly RDT rates and incidence: Additional details on the average monthly RDT rates and *P. falciparum* and *P. vivax* incidence by year and number of years open.

**Additional file 3.** Model-based predictions: Predictions of RDT rates by township and malaria post years open using negative binomial mixed modelling.

**Additional file 4.** RDT quality control results.

## Data Availability

The data analysed for this study are available upon request to the Mahidol-Oxford Tropical Medicine Research Unit data access committee: https://www.tropmedres.ac/units/moru-bangkok/bioethics-engagement/data-sharing.

## Abbreviations

METF: Malaria Elimination Task Force
RR: Rate Ratio
*P. falciparum*: *Plasmodium falciparum*
*P. vivax*: *Plasmodium vivax*
IPD: Inpatient department
RDT: Rapid diagnostic test
G6PD: Glucose-6-phosphate dehydrogenase
ICMW: Integrated community malaria worker
TB: Tuberculosis
HIV: Human immunodeficiency virus
STI: Sexually transmitted infections
IQR: Interquartile range
CI: Confidence interval
MAM: Medical Action Myanmar
