# Supplementary material for: Longitudinal trends in malaria testing rates in the face of elimination in eastern Myanmar: a 7-year observational study
Source: BMC Public Health. 2021 Sep 22;21:1725. doi: 10.1186/s12889-021-11749-x (PMC8459519; doi:10.1186/s12889-021-11749-x)
Supplement: Supplementary file 1 — Additional file 1. Incidence and RDT rate figures: Additional figures on incidence and RDT rates by date and malaria post time open. [file 12889_2021_11749_MOESM1_ESM.docx]

**Additional file 1 – Incidence and RDT rate figures**


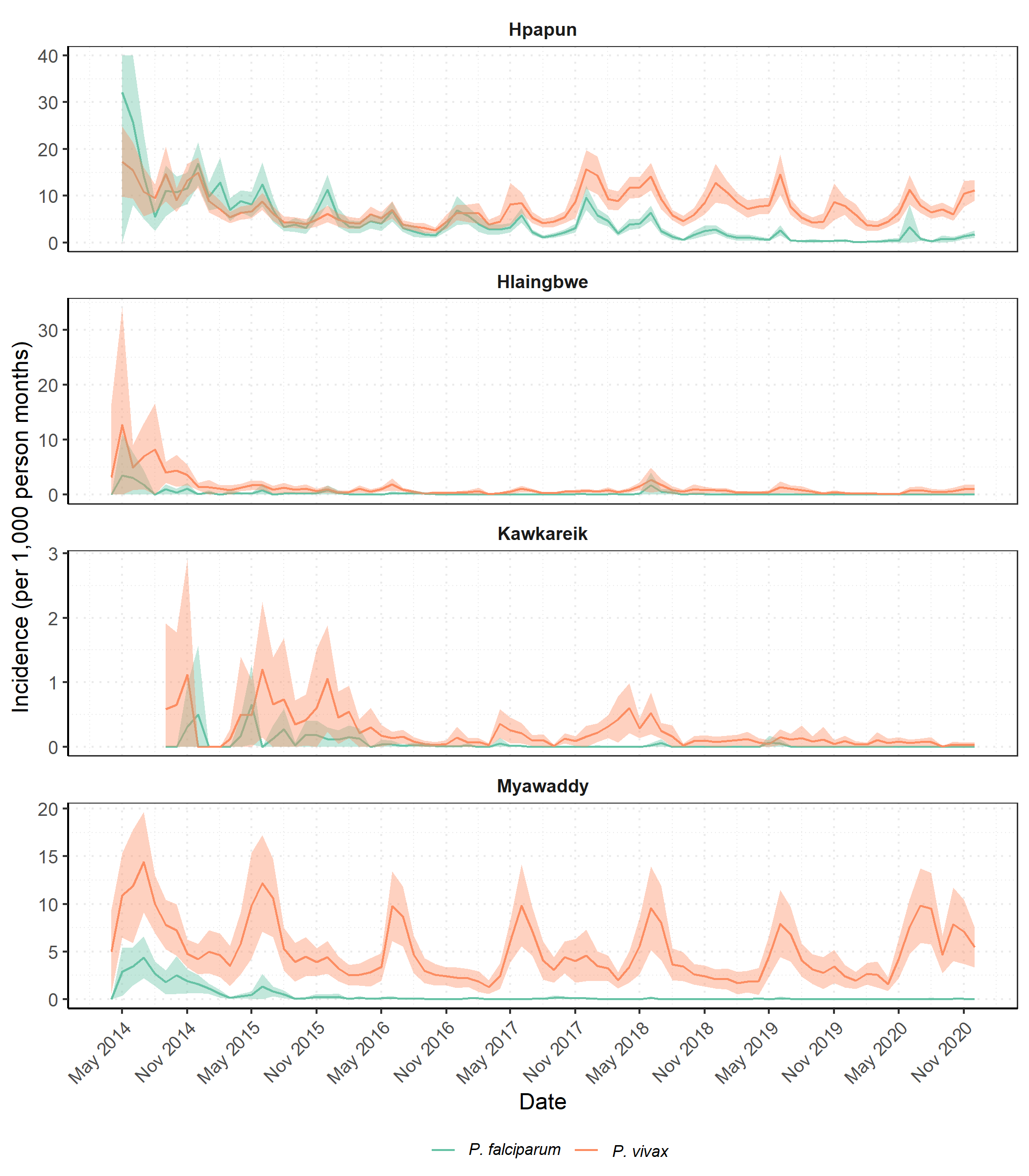


**Figure S1 Average monthly *P. falciparum* and *P. vivax* incidence at malaria posts by township.** Monthly incidence of *P. falciparum* (green line), with 95% confidence intervals (green area) and *P. vivax* (orange line), with 95% confidence intervals (orange area) calculated at the malaria post level, averaged over township. Upper confidence interval for *P. falciparum* incidence rate in Hpapun capped at 40. Different y-axes by township.


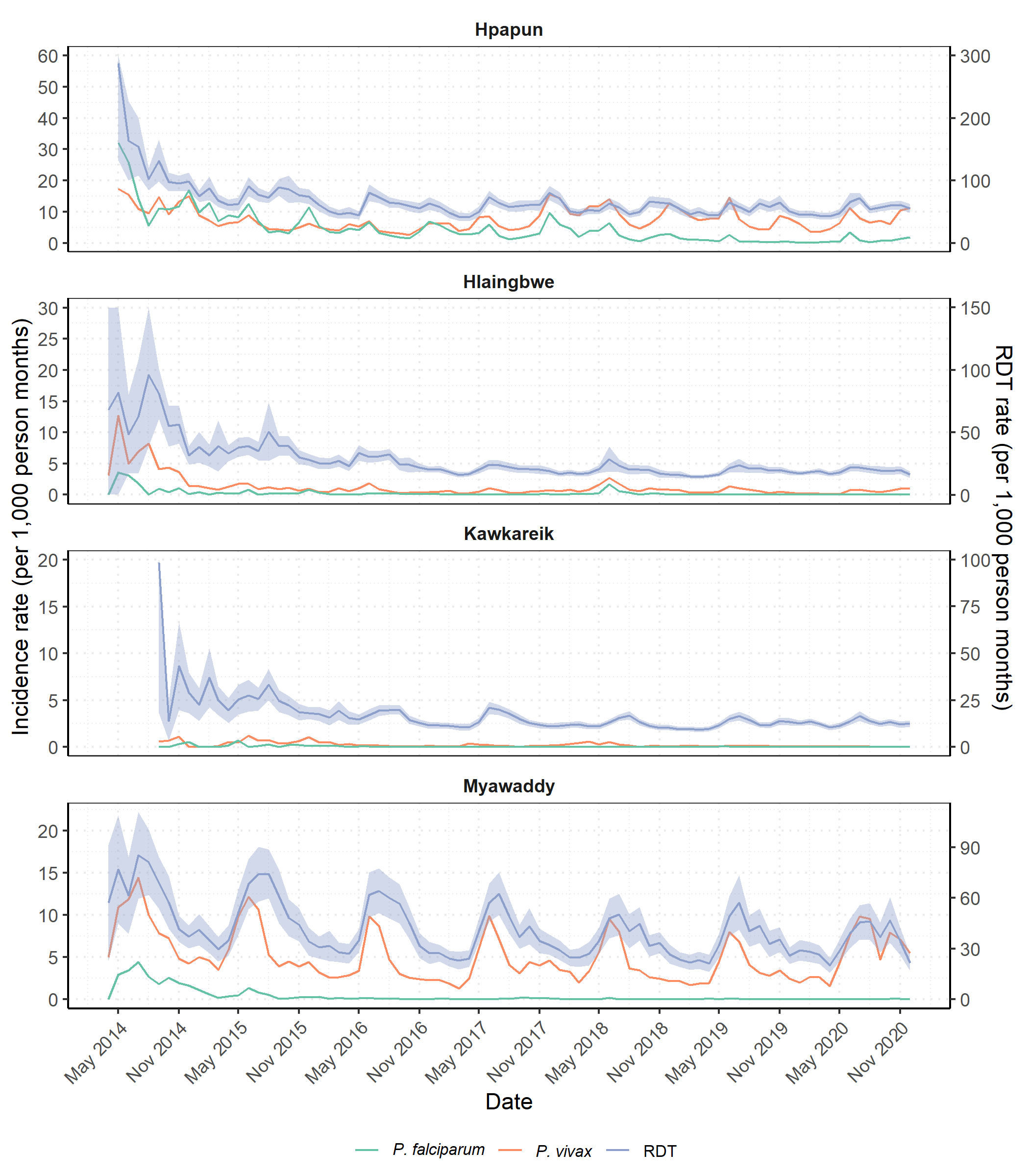


**Figure S2 Average monthly rate of RDTs, and malaria incidence by date and township.** Average rapid diagnostic testing rate (RDT – purple line), with 95% confidence intervals (purple area), and average *P. falciparum* (green line) and *P. vivax* (orange line) incidence rates in the METF malaria posts by date. Upper confidence interval for RDT rate in Hpapun capped at 300. Different y-axes by township.


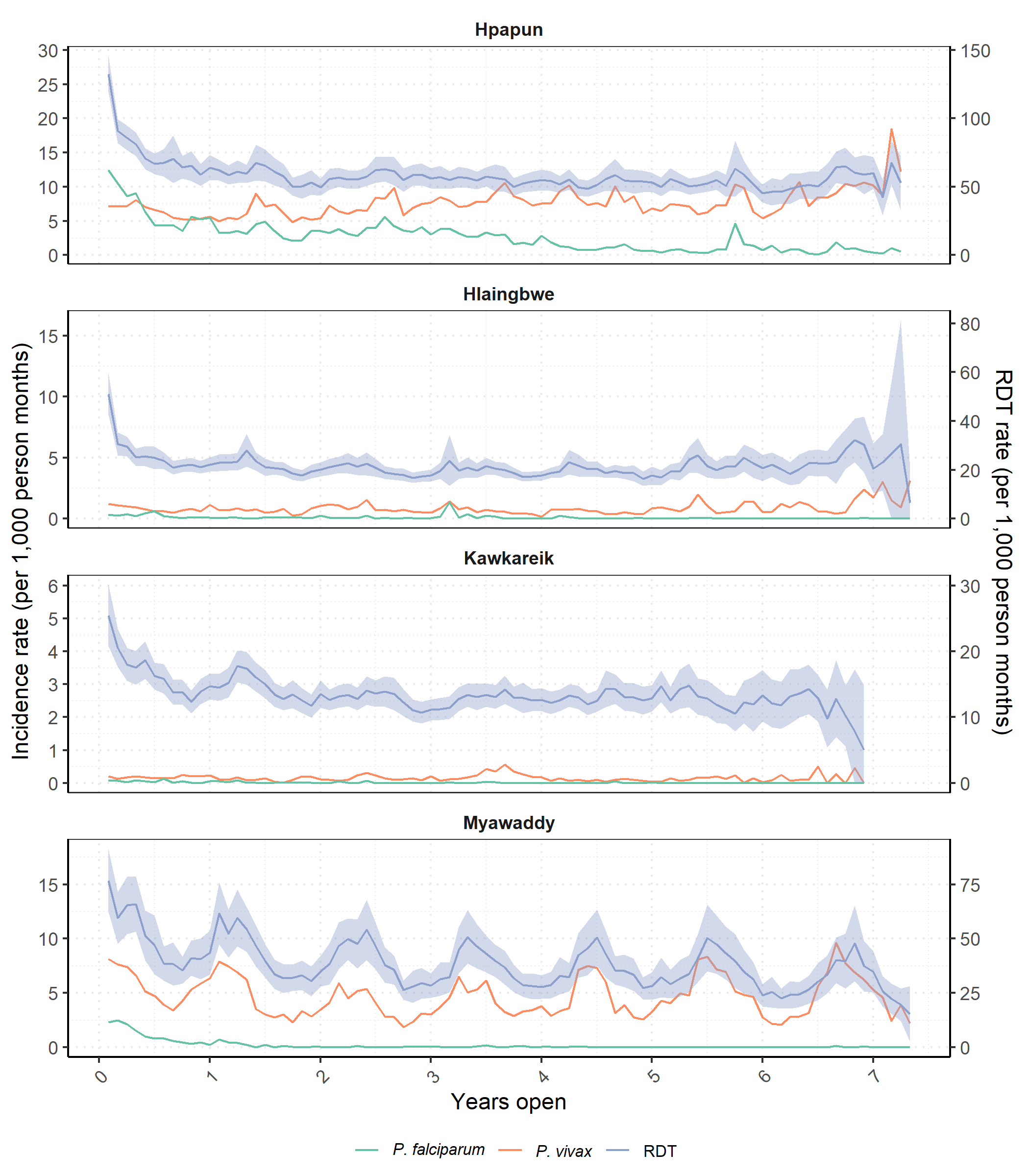


**Figure S3 Average monthly rate of RDTs, and malaria incidence by malaria post time open.** Average rapid diagnostic testing rate (RDT – purple line), with 95% confidence intervals (purple area), and average *P. falciparum* (green line) and *P. vivax* (orange line) incidence rates in the METF malaria posts by years open. Different y-axes by township.
