# Supplementary material for: Longitudinal trends in malaria testing rates in the face of elimination in eastern Myanmar: a 7-year observational study
Source: BMC Public Health. 2021 Sep 22;21:1725. doi: 10.1186/s12889-021-11749-x (PMC8459519; doi:10.1186/s12889-021-11749-x)
Supplement: Supplementary file 2 — Additional file 2. Monthly RDT rates and incidence: Additional details on the average monthly RDT rates and P. falciparum and P. vivax incidence by year and number of years open. [file 12889_2021_11749_MOESM2_ESM.docx]

**Additional file 2 — Monthly RDT rates and incidence.**

**Table S1 Number of MPs, average monthly RDT rate, and malaria incidence by township and calendar year.** Monthly malaria incidence calculated separately for *P. falciparum* and *P. vivax* malaria.

| Township | Year | Number of MP | RDT rate  per 1000 pmonths | *P. falciparum* incidence per 1000 pmonths | *P. vivax* incidence per 1000 pmonths |
| --- | --- | --- | --- | --- | --- |
| Hpapun | 2014 | 123 | 121.25 | 13.99 | 12.81 |
|  | 2015 | 365 | 77.31 | 7.41 | 5.80 |
|  | 2016 | 474 | 59.40 | 3.88 | 4.60 |
|  | 2017 | 478 | 57.91 | 3.71 | 6.81 |
|  | 2018 | 485 | 57.01 | 3.17 | 9.79 |
|  | 2019 | 487 | 54.03 | 0.87 | 7.92 |
|  | 2020 | 503 | 54.19 | 0.85 | 7.06 |
| Hlaingbwe | 2014 | 61 | 56.94 | 0.84 | 4.09 |
|  | 2015 | 159 | 36.59 | 0.34 | 1.16 |
|  | 2016 | 382 | 26.42 | 0.16 | 0.65 |
|  | 2017 | 390 | 20.20 | 0.05 | 0.54 |
|  | 2018 | 392 | 19.55 | 0.31 | 1.08 |
|  | 2019 | 387 | 18.47 | 0.01 | 0.61 |
|  | 2020 | 383 | 18.92 | 0.00 | 0.50 |
| Kawkareik | 2014 | 18 | 43.27 | 0.26 | 0.58 |
|  | 2015 | 62 | 24.63 | 0.16 | 0.55 |
|  | 2016 | 220 | 16.36 | 0.03 | 0.14 |
|  | 2017 | 220 | 13.82 | 0.01 | 0.13 |
|  | 2018 | 221 | 12.32 | 0.01 | 0.26 |
|  | 2019 | 232 | 12.21 | 0.01 | 0.10 |
|  | 2020 | 230 | 12.85 | 0.00 | 0.05 |
| Myawaddy | 2014 | 75 | 60.97 | 2.43 | 8.18 |
|  | 2015 | 101 | 49.92 | 0.45 | 6.02 |
|  | 2016 | 104 | 41.66 | 0.06 | 3.98 |
|  | 2017 | 105 | 37.84 | 0.06 | 4.23 |
|  | 2018 | 107 | 34.57 | 0.01 | 4.11 |
|  | 2019 | 107 | 33.73 | 0.02 | 3.54 |
|  | 2020 | 106 | 33.49 | 0.01 | 5.43 |

MP: Malaria post; RDT: Rapid diagnostic test; pmonths: person-months

**Table S2 Number of MPs, average monthly RDT rate, and malaria incidence rate by township year open.** Monthly malaria incidence calculated separately for *P. falciparum* and *P. vivax* malaria.

| Township | Year Open | Number of MPs | RDT rate per 1000 pmonths | *P. falciparum* incidence per 1000 pmonths | *P. vivax* incidence per 1000 pmonths |
| --- | --- | --- | --- | --- | --- |
| Hpapun | 1 | 507 | 79.21 | 6.81 | 6.35 |
|  | 2 | 489 | 57.59 | 3.37 | 6.19 |
|  | 3 | 486 | 57.65 | 3.78 | 7.42 |
|  | 4 | 479 | 54.05 | 2.18 | 8.40 |
|  | 5 | 471 | 53.02 | 0.80 | 7.39 |
|  | 6 | 361 | 52.06 | 1.14 | 7.52 |
|  | 7 | 120 | 62.12 | 0.88 | 10.33 |
| Hlaingbwe | 1 | 400 | 27.16 | 0.24 | 0.83 |
|  | 2 | 395 | 21.47 | 0.10 | 0.73 |
|  | 3 | 382 | 19.90 | 0.17 | 0.82 |
|  | 4 | 373 | 19.59 | 0.11 | 0.59 |
|  | 5 | 356 | 19.41 | 0.02 | 0.61 |
|  | 6 | 153 | 21.63 | 0.02 | 0.99 |
|  | 7 | 59 | 25.86 | 0.01 | 1.17 |
| Kawkareik | 1 | 234 | 16.91 | 0.05 | 0.17 |
|  | 2 | 234 | 14.24 | 0.02 | 0.11 |
|  | 3 | 220 | 12.44 | 0.01 | 0.15 |
|  | 4 | 217 | 13.04 | 0.01 | 0.24 |
|  | 5 | 215 | 13.25 | 0.01 | 0.09 |
|  | 6 | 62 | 12.51 | 0.00 | 0.14 |
|  | 7 | 17 | 9.56 | 0.00 | 0.15 |
| Myawaddy | 1 | 109 | 51.37 | 1.12 | 5.86 |
|  | 2 | 109 | 41.65 | 0.17 | 4.31 |
|  | 3 | 108 | 37.81 | 0.04 | 3.86 |
|  | 4 | 13 | 35.75 | 0.06 | 4.10 |
|  | 5 | 102 | 35.79 | 0.01 | 4.70 |
|  | 6 | 98 | 34.45 | 0.00 | 4.82 |
|  | 7 | 72 | 36.53 | 0.03 | 6.55 |

MP: Malaria post; RDT: Rapid diagnostic test; pmonths: person-months
