# Supplementary material for: Longitudinal trends in malaria testing rates in the face of elimination in eastern Myanmar: a 7-year observational study
Source: BMC Public Health. 2021 Sep 22;21:1725. doi: 10.1186/s12889-021-11749-x (PMC8459519; doi:10.1186/s12889-021-11749-x)
Supplement: Supplementary file 3 — Additional file 3. Model-based predictions: Predictions of RDT rates by township and malaria post years open using negative binomial mixed modelling. [file 12889_2021_11749_MOESM3_ESM.docx]

**Additional file 3 — Model-based predictions for RDT rates by township and malaria post years open.**


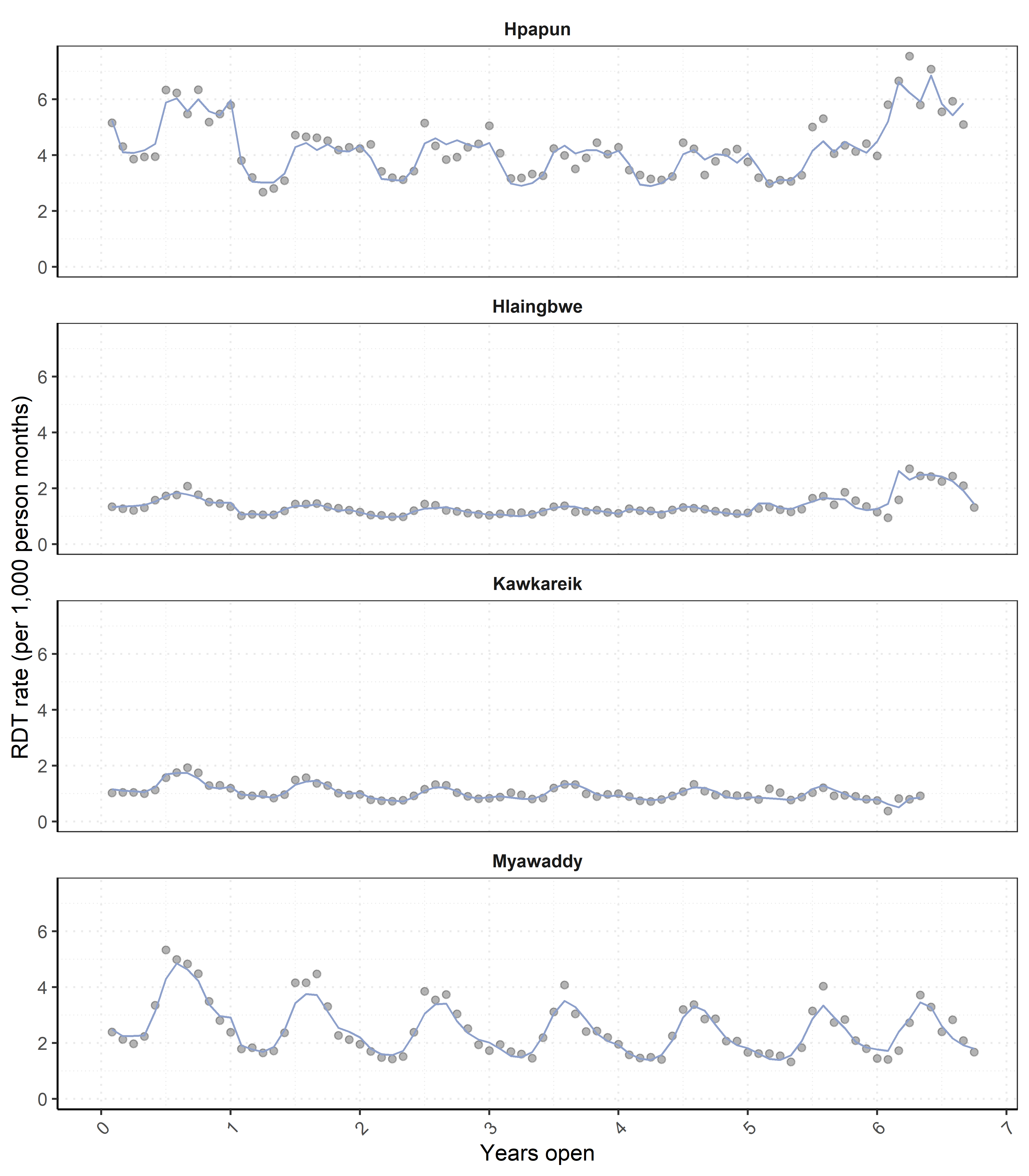


**Figure S4 Model-based predictions of the monthly RDT rate by malaria post years open.** Predictions from a negative binomial mixed-effects model (blue line) alongside aggregated observed monthly RDT rates (grey points).
