# Supplementary material for: Longitudinal trends in malaria testing rates in the face of elimination in eastern Myanmar: a 7-year observational study
Source: BMC Public Health. 2021 Sep 22;21:1725. doi: 10.1186/s12889-021-11749-x (PMC8459519; doi:10.1186/s12889-021-11749-x)
Supplement: Supplementary file 4 — Additional file 4. RDT quality control results. [file 12889_2021_11749_MOESM4_ESM.docx]

**Additional file 4 — RDT quality control results.**

**Table S3 RDT quality control results by township and years.**

| Township | Year | RDTs checked | Damaged;  n(%) | Record;  n(%) | User;  n(%) |
| --- | --- | --- | --- | --- | --- |
| Hpapun | 2016 | 1409 | 115 (8.16) | 156 (11.07) | 214 (15.19) |
|  | 2017 | 2288 | 148 (6.47) | 121 (5.29) | 224 (9.79) |
|  | 2018 | 1439 | 91 (6.32) | 123 (8.55) | 146 (10.15) |
|  | 2019 | 782 | 22 (2.81) | 29 (3.71) | 98 (12.53) |
|  | 2020 | 357 | 22 (6.16) | 10 (2.80) | 23 (6.44) |
| Hlaingbwe | 2016 | 427 | 13 (3.04) | 45 (10.54) | 65 (15.22) |
|  | 2017 | 492 | 17 (3.46) | 92 (18.70) | 54 (10.98) |
|  | 2018 | 398 | 11 (2.76) | 37 (9.30) | 43 (10.80) |
|  | 2019 | 164 | 5 (3.05) | 11 (6.71) | 11 (6.71) |
|  | 2020 | 99 | 4 (4.04) | 7 (7.07) | 6 (6.06) |
| Kawkareik | 2016 | 430 | 4 (0.93) | 74 (17.21) | 69 (16.05) |
|  | 2017 | 360 | 8 (2.22) | 30 (8.33) | 35 (9.72) |
|  | 2018 | 373 | 15 (4.02) | 37 (9.92) | 50 (13.40) |
|  | 2019 | 256 | 6 (2.34) | 9 (3.52) | 27 (10.55) |
|  | 2020 | 193 | 3 (1.55) | 2 (1.04) | 21 (10.88) |
| Myawaddy | 2016 | 856 | 65 (7.59) | 89 (10.40) | 75 (8.76) |
|  | 2017 | 698 | 16 (2.29) | 69 (9.89) | 50 (7.16) |
|  | 2018 | 188 | 2 (1.06) | 7 (3.72) | 14 (7.45) |
|  | 2019 | 314 | 2 (0.64) | 25 (7.96) | 34 (10.83) |
|  | 2020 | 75 | 0 (0.00) | 3 (4.00) | 15 (20.00) |

Damaged: scratched, dirty test strip and/or ink on strip.

Record: improper or no recording of test result and/or time test run.

User: blood on side of well, or blood did not migrate, but test result recorded.
